# On the characterization of claw-free graphs with given total restrained domination number

**DOI:** 10.1186/s40064-016-3387-7

**Published:** 2016-10-07

**Authors:** Xiaoming Pi

**Affiliations:** Department of Mathematics, Harbin Normal University, Harbin, 150025 China

**Keywords:** Claw-free graphs, Total restrained dominating set, Total restrained domination number, 05C69

## Abstract

A set *S* of vertices in graph $G=(V,E)$ is a $total\, restrained\, dominating\, set$, abbreviated TRDS, of *G* if every vertex of *G* is adjacent to a vertex in *S* and every vertex of $V-S$ is adjacent to a vertex in $V-S$. The $total\, restrained\, domination\, number$ of *G*, denoted by $\gamma _{tr}(G)$, is the minimum cardinality of a TRDS of *G*. Jiang and Kang (J Comb Optim. 19:60–68, 2010) characterized the connected claw-free graph *G* of order *n* with $\gamma _{tr}(G)=n$. This paper studies the total restrained domination number of claw-free graphs and characterizes the connected claw-free graph *G* of order *n* with $\gamma _{tr}(G)=n-2$.

## Background

Let $G=(V,E)$ be a simple graph with vertex set *V* and edge set *E*. For a vertex *u* of *V*, let $N_{G}(u)=\{v\in V|uv\in E\}$ denote the ${open}\, {neighborhood}$ of *u* and $N_{G}[u]=N(u)\cup \{u\}$ denote the ${closed}\, {neighborhood}$ of *u*. The *degree* of *u* is denoted by $d_G(u)$ (briefly *N*(*u*), *N*[*u*], *d*(*u*) when no ambiguity on the graph is possible). For a subset *S* of *V*, let *G*[*S*] be the subgraph of *G* induced by *S*. A vertex *v* is called a $support\, vertex$ of *G* if *v* is adjacent to a vertex of degree one. A support vertex is *strong* if it is adjacent to at least two vertices of degree one. Let *S*(*G*) and *L*(*G*) denote the set of all support vertices and all vertices of degree one in *G*, respectively. An edge is called a $pendant\, edge$ if it is incident with a vertex of degree one. Let $\omega (G)$ denote the number of components of *G*. The *corona* $H\circ K_1$ of a graph *H* is the graph obtained from *H* by attaching a pendant edge to each vertex of *H*. A cycle of order *k* is denoted by $C_k$. A graph is *claw*-*free* if it contains no $K_{1,3}$ as an induced subgraph. A set *S* of vertices in graph $G=(V,E)$ is a $total\, restrained\, dominating\, set$, abbreviated TRDS, of *G* if every vertex of *G* is adjacent to a vertex in *S* and every vertex of $V-S$ is adjacent to a vertex in $V-S$. The $total\, restrained\, domination\, number$ of *G*, denoted by $\gamma _{tr}(G)$, is the minimum cardinality of a TRDS of *G*. A TRDS of cardinality $\gamma _{tr}(G)$ is called a $\gamma _{tr}$-set. For other notations and graph theory terminologies we in general follow Haynes et al. (1998).

The concept of total restrained domination in graphs was introduced in Haynes et al. (1998), albeit indirectly, as a vertex partitioning problem and has been studied, in Telle and Proskurowski (1997), Cyman and Raczek (2006), Dankelmann et al. (2006), Hattingh et al. (2007), Henning and Maritz (2008), Ma et al. (2005), Raczek (2007), Raczek and Cyman (2008), Zelinka (2005) and Jiang and Kang (2010). Jiang and Kang (2010) characterized the connected claw-free graph *G* of order *n* with $\gamma _{tr}(G)=n$.

This paper characterizes the connected claw-free graph *G* of order *n* with $\gamma _{tr}(G)=n-2$.

## Main results

### **Lemma 1**

(Jiang and Kang 2010) *Let* *G* *be a connected claw-free graph with order* $n\ge 2$. *Then* $\gamma _{tr}(G)=n$ *if and only if* $G\in \Gamma$, *where* $\Gamma =\cup _{i=0}^3\Gamma _i$, $\Gamma _0=\{G|G$ *is the corona* $K_m\circ K_1$ *of* $K_m,m\ge 1\}$, $\Gamma _1$ *is a collection of all graphs obtained from* $G'\in \Gamma _0$ *by subdividing exactly one pendant edge*, $\Gamma _2$ *is a collection of all graphs obtained from* $G'\in \Gamma _0$ *by adding a new vertex and joining it to all the support vertices of* $G'$, *and* $\Gamma _3$ *is a collection of all graphs obtained from* $G_1,G_2\in \Gamma _0 (|V(G_1)|,|V(G_2)|\ge 3)$ *by adding a new vertex* *u* *and joining it to all the support vertices of graphs* $G_1$ *and* $G_2$.

For completing our characterization, we define a family $\Gamma ^2$ of claw-free graphs as follows.

- $\Gamma _0^2$ is a collection of all graphs obtained from $G_1,G_2\in \Gamma _0$ by joining $G_1,G_2$ with an edge *uv*, where $u\in L(G_1)$, $v\in L(G_2)$ and $|V(G_1)|,|V(G_2)|\ge 4$.
- $\Gamma _1^2$ is a collection of all graphs obtained from $G_1,G_2\in \Gamma _0$ by joining $G_1,G_2$ with a path $P_3=(u,v,x)$, where $u\in L(G_1)$, $x\in L(G_2)$, $|V(G_1)|,|V(G_2)|\ge 4$.
- $\Gamma _2^2$ is a collection of all graphs obtained from $G_1\in \Gamma _0-\{K_2\}$ and $K_3$ by adding a new edge *uv*, where $u\in L(G_1)$ and $v\in V(K_3)$.
- $\Gamma _3^2$ is a collection of all graphs obtained from $G_1,G_2\in \Gamma _2$ by joining $G_1,G_2$ with an edge *uv*, where $u\in V(G_1)-S(G_1)-L(G_1)$, $v\in V(G_2)-S(G_2)-L(G_2)$ and $|V(G_1)|,|V(G_2)|\ge 5$.
- $\Gamma _4^2$ is a collection of all graphs obtained from $G_1, G_2\in \Gamma _0$ by uniting two vertices *u* and *v*, where $u\in L(G_1)$, $v\in L(G_2)$ and $|V(G_1)|,|V(G_2)|\ge 4$.
- $\Gamma _5^2$ is a collection of all graphs obtained from $G'\in \Gamma -\Gamma _0$ by attaching a pendant edge to a vertex of $L(G')$, where $|V(G')|\ge 5$.
- $\Gamma _6^2$ is a collection of a 5-cycle, a 6-cycle, a 7-cycle and all graphs obtained from $G'\in \Gamma _0$ by adding an edge *uv*, where $u,v\in L(G')$ and $|V(G')|\ge 4$.
- $\Gamma _7^2$ is a collection of all graphs obtained from $G'\in \Gamma -(\Gamma _0\cup \{P_3\})$ by adding a new vertex and joining it to every vertex of $V(G')-L(G')$.
- $\Gamma _8^2$ is a collection of all graphs obtained from $G_1\in \Gamma _2\cup \Gamma _3,G_2\in \Gamma _0$ by deleting a vertex *u* of $L(G_1)$ and joining $u'$ to every vertex of $S(G_2)$, where $u'\in N_{G_1}(u)$ and $|V(G_1)|\ge 5$.
- $\Gamma _{9}^2$ is a collection of all graphs obtained from $G'\in \Gamma _1$ by adding a new vertex and joining it to every vertex of *N*[*u*], where *u* is a 2-degree vertex in $S(G')$.
- $\Gamma _{10}^2$ is a collection of all graphs obtained from $G_1,G_2\in \Gamma _0$ by adding a new vertex and joining it to each endpoint of a pendant edge of $G_1$ and every vertex of $S(G_2)$ or from $G_1\in \Gamma _0,K_2$ by adding a new vertex and joining it to each endpoint of a pendant edge of $G_1$ and one vertex of $K_2$.
- $\Gamma _{11}^2$ is a collection of all graphs obtained from $G'\in \Gamma _0$ by adding a new vertex and joining it to every vertex of $S(G')$ and a vertex of $L(G')$, where $|V(G')|\ge 4$.
- $\Gamma _{12}^2$ is a collection of all graphs obtained from $G'\in \Gamma _1$ by adding a new vertex and joining it to each endpoint of a pendant edge of $G'$.

Let $$\Gamma ^2=\cup _{i=0}^{12}\Gamma _i^2$$.

### **Theorem 1**

*Let* *G* *be a connected claw-free graph of order* $n\ge 4$. *Then* $\gamma _{tr}(G)=n-2$ *if and only if* $G\in \Gamma ^2$.

### Proof

Clearly when $G\in \Gamma ^2$, $\gamma _{tr}(G)=n-2$. Let *G* be a connected claw-free graph of order *n* with $\gamma _{tr}(G)=n-2$ and *S* be a $\gamma _{tr}$-set of *G*. Let $V-S=\{v_1,v_2\}$. Clearly, *G*[*S*] has at most three components. $\square$

### Claim 1

Let *C* be a component of *G*[*S*]. If $N(v_i)\cap V(C)=\{v'\},\,i=1,2$, then $G[N_C[v']]\simeq K_{d_C(v')}$.

### Proof

It is obvious. $\square$

### Claim 2

Let $C,C'$ be two components of *G*[*S*]. If $d(v_1)=d(v_2)=2$, $N(v_1)\cap V(C)=\{v'\}$ and $N(v_2)\cap V(C')=\{v''\}$, then $G\in \Gamma _0^2\cup \Gamma _1^2\cup \Gamma _2^2\cup \Gamma _4^2\cup \Gamma _5^2$.

### Proof

Let $C,C'\ne K_2$. Then by Claim 1, $v'\not \in S(C),v''\not \in S(C')$. Denote $G'=G[V(C)\cup \{v_1\}]$ and $G''=G[V(C')\cup \{v_2\}]$. Assume that $\gamma _{tr}(G')\le |V(C)|-1$ and $S'$ is a $\gamma _{tr}$-set of $G'$. Since $v_1$ is a 1-degree vertex of $G'$, $v_1,v'\in S'$. Consequently $S'\cup (V(C')-\{v''\})$ is a TRDS of *G*, which is a contradiction. Hence $\gamma _{tr}(G')=|V(C)|+1$ and by Lemma 1, $G'\in \Gamma$. By the same reason, $G''\in \Gamma$. Obviously, $G',G''\not \in \Gamma _2\cup \Gamma _3$. Let $G'\in \Gamma _1$. Assume that $d(v')\ne 2$. Then $G'\ne P_5$. Let *u* be the 2-degree vertex in $S(G')$ and $u'$ be the neighbor of *u* not in $L(G')$. Then $V(G)-\{u',v',v_1\}$ is a TRDS of *G*, which is a contradiction. Hence $d(v')=2$. By the same reason, when $G''\in \Gamma _1$, $d(v'')=2$. Clearly, at most one of $G',G''$ is in $\Gamma _1$. When $G', G''\in \Gamma _0$, $G\in \Gamma _0^2$. When one of $G', G''$ is in $\Gamma _1$, $G\in \Gamma _1^2$.

Let one of $C,C'$ be isomorphic to $K_2$. Without loss of generality, let $C'=K_2$. Assume that $C\not \in \Gamma$ and $S'$ is a $\gamma _{tr}$-set of *C*. Then $|S'|\le |V(C)|-2$. If $v'\in S'$, then $S'\cup V(C')$ is a TRDS of *G*, which is a contradiction. Hence $v'\not \in S'$. However, $S'\cup V(C')\cup \{v_2\}$ is a TRDS of *G*, which is a contradiction. Thus $C\in \Gamma$. By Claim 1, when $C\ne K_2$, $v'\not \in S(C)$. Assume that $C\in \Gamma _3$. By the claw-freeness of *G*, $v'\in L(C)$ and $V(G)-\{v',x,y\}$ is a TRDS of *G*, where $x\in N_C(v')$ and $y\in V(C)-S(C)-L(C)$. It’s a contradiction. So $C\not \in \Gamma _3$. When $C\in \Gamma _0$, $v'\in L(C)$ and $G\in \Gamma _0^2\cup \{P_6\}\subseteq \Gamma _0^2\cup \Gamma _5^2$. When $C\in \Gamma _1$, we also have $v'\in L(C)$. Let $u\in N_C(v')$. Then *u* is the vertex with minimum degree in *S*(*C*). Therefore $G\in \Gamma _1^2\cup \{P_7\}\subseteq \Gamma _1^2\cup \Gamma _4^2$. When $C\in \Gamma _2$, $v'\in V(C)-S(C)-L(C)$ and $G\in \Gamma _2^2\cup \Gamma _4^2$. $\square$

### Claim 3

Let *C* be a component of *G*[*S*]. If $d=|N(v_i)\cap V(C)|\ge 2, i=1,2$, then $G[N(v_i)\cap V(C)]\simeq K_d$.

### Proof

Without loss of generality, let $d=|N(v_1)\cap V(C)|\ge 2$. Assume that $u_1,u_2\in N(v_1)\cap V(C)$ and $u_1u_2\not \in E(G)$. By the claw-freeness of *G*, either $v_2u_1\in E(G)$ or $v_2u_2\in E(G)$. Without loss of generality, let $v_2u_1\in E(G)$. By the connectivity of *C*, there is a shortest path $P=(u_1=w_1,w_2,\ldots ,w_p=u_2)(p\ge 3)$ joining $u_1$ to $u_2$ in *C*. If $u_2\not \in S(C)$, then we get a contradiction that $S-\{u_2\}$ is a TRDS of *G*. Hence $u_2\in S(C)$. Let $u'\in N_C(u_2)\cap L(C)$. By the claw-freeness of *G*, $v_1w_{p-1}\in E(G)$ or $v_1u'\in E(G)$. If $v_1u'\in E(G)$, then $S-\{u'\}$ is a TRDS of *G*, which is a contradiction. Hence $v_1w_{p-1}\in E(G)$. By the same reason, $w_{p-1}\in S(C)$. If $w_{p-2}\in N_C(w_{p-1})\cap L(C)$, then $u_1=w_{p-2}$ and $(S-\{u_1,w_{p-1}\})\cup \{v_2\}$ is a TRDS of *G*, which is a contradiction. So $w_{p-2}\not \in N_C(w_{p-1})\cap L(C)$. Let $u''\in N_C(w_{p-1})\cap L(C)$. Then $G[\{u_2,w_{p-1},w_{p-2},u''\}]\simeq K_{1,3}$, which is a contradiction. So $u_1u_2\in E(G)$ and $G[N(v_1)\cap V(C)]\simeq K_d$. $\square$

### Claim 4

Let *C* be a component of *G*[*S*] and $|N(v_1)\cap V(C)|\ge 2$. If $|N(v_2)\cap V(C)|=1$, then (1)$\omega (G[S])\le 2$ and when $|V(C)|\ge 3$, $C=G[S]$; (2)$C\in \{P_3\}\cup \Gamma _0\cup \Gamma _2-\{K_3\}$ and $G\in \Gamma _6^2\cup \Gamma _{9}^2\cup \Gamma _{11}^2$.

### Proof

Let $u\in N(v_2)\cap V(C)$. We affirm that $N(v_1)-\{v_2\}\subseteq V(C)$. Otherwise, by the claw-freeness *G*, $N(v_1)\cap (S-V(C))\subseteq N(v_2)\cap (S-V(C))$. When $|V(C)|=2$, we get the contradiction that $(S-V(C))\cup \{v_1\}$ is a TRDS of *G*; when $|V(C)|\ge 3$, by Claim 1, $u\not \in S(C)$ and we get the contradiction that $S-\{u\}$ is a TRDS of *G*.

1. By the claw-freeness of *G*, at most two components of *G*[*S*] contain vertices of $N(v_2)-\{v_1\}$. It follows that $\omega (G[S])\le 2$. Let $|V(C)|\ge 3$. Assume that $\omega (G[S])=2$ and $C'$ is a component of *G*[*S*] other than *C*. Then $N(v_2)\cap V(C')\ne \emptyset$. By Claim 1 and $|V(C)|\ge 3$, $u\not \in S(C)$. Therefore $S-\{u\}$ is a TRDS of *G*, which is a contradiction. Hence *G*[*S*] is connected.
2. Let $|V(C)|=2$. If $C=G[S]$, then the result holds. Assume that $\omega (G[S])=2$ and $C'$ is the other component of *G*[*S*]. By above, $N(v_1)\cap V(C')=\emptyset$. If $N(v_2)\cap V(C')\not \subseteq S(C')$ and $u'$ is a neighbor of $v_2$ in $V(C')-S(C')$, then $C'\ne K_2$ and $S-\{u'\}$ also is a TRDS of *G*, which is a contradiction. Hence $N(v_2)\cap V(C')\subseteq S(C')$. By the connectivity of $C'$ and the claw-freeness of *G*, we have $V(C')-N(v_2)-L(C')=\emptyset$. By Claim 1 and Claim 3, $G[N(v_2)\cap V(C')]$ is a complete graph and $C'\in \Gamma _0$. Clearly, $G[V(C')\cup \{v_2\}]\ne K_3$. Thus $G\in \Gamma _9^2$.

Let $|V(C)|\ge 3$. By (1), $C=G[S]$. Assume that $C\not \in \Gamma$ and $S'$ is a $\gamma _{tr}$-set of *C*. Clearly $\gamma _{tr}(C)=|V(C)|-2$. Let $u',u''$ be in $V(C)-S'$. Then $u'u''\in E(G)$ and at least one of $N(v_1)\cap S', N(v_2)\cap S'$ is empty. If $N(v_1)\cap S'=\emptyset$ and $N(v_2)\cap S'\ne \emptyset$ (or $N(v_1)\cap S'\ne \emptyset$ and $N(v_2)\cap S'=\emptyset$), then $S'\cup \{v_2\}$ (or $S'\cup \{v_1\}$) is a TRDS of *G*, which is a contradiction. Thus $N(v_1)\cap S'=N(v_2)\cap S'=\emptyset$. Then $(N(v_1)\cup N(v_2))\cap V(C)=\{u',u''\}$. Let $u'=u\in N(v_1)\cap N(v_2)$. Then $S'\cup \{u'\}$ is a TRDS of *G*, a contradiction. So $C\in \Gamma$.

Clearly $C\ne K_3$. Assume that $C\in \Gamma -(\{P_3\}\cup \Gamma _0\cup \Gamma _2)$. By claw-freeness of *G* and $|N(v_2)\cap V(C)|=1$, $u\in L(C)$. Let $u'\in N_C(u)$ and $u''\in V(C)-S(C)-L(C)$. If $v_1u'\in E(G)$, then $V(G)-\{v_1,u',u''\}$ is a TRDS of *G*, which is a contradiction. Hence $v_1u'\not \in E(G)$. It follows that $v_1u\not \in E(G)$. Otherwise since $v_1$ has a neighbor other than *u*, $V(G)-\{v_2,u,u'\}$ is a TRDS of *G*, which is a contradiction. If $d(u')\ge 3$, then we have the contradiction that $V(G)-\{u,u',u''\}$ is a TRDS of *G*. So $d(u')=2$ and $C\in \Gamma _1$. Assume that $v_1$ is adjacent to a vertex *v* of $L(C)-\{u\}$. Then by the claw-freeness of *G*, $v_1v'\in G$, where $v'\in N_C(v)$. However, $V(G)-\{v,v',u''\}$ is a TRDS of *G*, a contradiction. Hence $N(v_1)\cap L(C)=\emptyset$. By the claw-freeness, Claim 3 and $v_1u'\not \in G$, $v_1$ is adjacent to every vertex of $V(C)-(L(C)\cup \{u'\})$. However, $V(G)-\{u',u'',v_1\}$ is a TRDS of *G*, which is a contradiction. Hence $C\in \{P_3\}\cup \Gamma _0\cup \Gamma _2-\{K_2,K_3\}$.

When $C=P_3$, it is easy to check that $G\in \Gamma _{11}^2$. Let $C\ne P_3$. Clearly $u\not \in S(C)$. Let $u\in L(C)$ and $u'\in N_C(u)$. Assume that $C\in \Gamma _2-\{K_3\}$. Let $v'$ be the vertex in $V(C)-S(C)-L(C)$. Then $V(G)-\{u,u',v'\}$ is a TRDS of *G*, which is a contradiction. Hence $C\in \Gamma _0$. (a) When $v_1$ is adjacent to *u*. By Claim 3, $N(v_1)\cap V(C)=\{u,u'\}$ and $G\in \Gamma _9^2$. (b) When $v_1$ isn’t adjacent to *u*. If $v_1$ is adjacent to a vertex *v* of *L*(*C*), then by the claw-freeness of *G*, $v_1$ must be adjacent to $v'\in N_C(v)$. Therefore $V(G)-\{v,v',u'\}$ is a TRDS of *G*, which is a contradiction. Hence $N(v_1)\cap L(C)=\emptyset$ and $N(v_1)\cap V(C)\subseteq S(C)$. By the claw-freeness of *G*, $N(v_1)\cap V(C)=S(C)$. Therefore $G\in \Gamma _6^2$. Let $u\in V(C)-S(C)-L(C)$. Then $C\in \Gamma _2-\{K_3\}$. If $v_1$ is adjacent to a vertex *v* of *L*(*C*), then by Claim 3, $v_1$ must be adjacent to $v'\in N_C(v)$. Hence $S-\{v\}$ is a TRDS of *G*, which is a contradiction. Thus $N(v_1)\cap V(C)\subseteq V(C)-L(C)$. By the claw-freeness of *G* and $|N(v_1)\cap V(C)|\ge 2$, $N(v_1)\cap V(C)=V(C)-L(C)$ and $G\in \Gamma _{11}^2$. $\square$

### Claim 5

Let $C,C'$ be two components of *G*[*S*]. If $N(v_1)\cap V(C)=\{v'\}$, $N(v_1)\cap V(C')=\emptyset$, $N(v_2)\cap V(C)=\emptyset$ and $|N(v_2)\cap V(C')|\ge 2$, then $C\in \Gamma -\Gamma _3$, $G[V(C')\cup \{v_2\}]\in \Gamma _2$ and $G\in \Gamma _0^2\cup \Gamma _1^2\cup \Gamma _2^2\cup \Gamma _4^2\cup \Gamma _5^2\cup \Gamma _{12}^2$.

### Proof

If $|V(C')|=2$, then $C'=K_2$ and $G[\{v_2\}\cup V(C')]=K_3\in \Gamma _2$. Let $|V(C')|\ge 3$. If there is a vertex *v* in $N(v_2)\cap V(C')$ with degree $|N(v_2)\cap V(C')|$, then by Claims 1 and 3, $S-\{v\}$ is a TRDS of *G*, which is a contradiction. Hence for every vertex *v* of $N(v_2)\cap V(C')$, $d(v)\ge |N(v_2)\cap V(C')|+1$. If there is a vertex *v* in $N(v_2)\cap V(C')-S(C')$, then $S-\{v\}$ is a TRDS of *G*, which is a contradiction. Thus $N(v_2)\cap V(C')\subseteq S(C')$. By the claw-freeness of *G*, $N(v_2)\cap V(C')=S(C')$ and $G[V(C')\cup \{v_2\}]\in \Gamma _2$.

Assume that $C\not \in \Gamma$ and $S'$ is a $\gamma _{tr}$-set of *C*. Then $|S'|\le |V(C)|-2$. If $v'\in S'$, then $S'\cup (S-V(C))$ is a TRDS of *G*, which is a contradiction. Hence $v'\not \in S'$. However, $S'\cup (S-V(C))\cup \{v_2\}$ is a TRDS of *G*, which also is a contradiction. Thus $C\in \Gamma$. By Claim 1, when $C\not \simeq K_2$, $v'\not \in S(C)$. Assume that $C\in \Gamma _3$. Then by the claw-freeness of *G*, $v'\in L(C)$. Let *v* be the vertex in $V(C)-S(C)-L(C)$. Let $v''$ be the common neighbor of $v'$ and *v*, then $V(G)-\{v,v',v''\}$ is a TRDS of *G*, which is a contradiction. So $C\in \Gamma -\Gamma _3$.

By the claw-freeness of *G*, when $C\in \Gamma _0\cup \Gamma _1$, $v'\in L(C)$. When $G[V(C')\cup \{v_2\}]=K_3$, $C\in \{K_2, P_3\}\cup (\Gamma _2-\{K_3\})$. Otherwise, if $C\in (\Gamma _0-\{K_2\})\cup (\Gamma _1-\{P_3\})$ and $v''$ is the neighbor of $v'$ in *V*(*C*), then $V(G)-(\{v',v''\}\cup V(C'))$ is a TRDS of *G*, which is a contradiction; if $C=K_3$, then $\{v',v_1,v_2\}$ is a TRDS of *G*, which also is a contradiction.

Let *G*[*S*] has exactly two components. When $C\in \Gamma _0$, if $G[V(C')\cup \{v_2\}]=K_3$, then $C=K_2$ and $G\in \Gamma _{12}^2$; if $G[V(C')\cup \{v_2\}]\ne K_3$, then when $C=K_2$, $G\in \Gamma _5^2$, and when $C\ne K_2$, $G\in \Gamma _0^2$. Let $C\in \Gamma _1$. If $C=P_3$, then clearly $C-\{v'\}\in \Gamma _0$. Let $C\ne P_3$, $v''$ be the vertex in $V(C)-S(C)-L(C)$ and $v'''$ be the common neighbor of $v',v''$. If $C-\{v'\}\not \in \Gamma _0$, then $V(G)-\{v',v'',v'''\}$ is a TRDS of *G*, which is a contradiction. Hence $C-v'\in \Gamma _0$. When $C=P_3$ and $G[V(C')\cup \{v_2\}]=K_3$, $G\in \Gamma _2^2$; when $G[V(C')\cup \{v_2\}]\ne K_3$, $G\in \Gamma _1^2$; when $C\in \Gamma _1-P_3$, $G[V(C')\cup \{v_2\}]\ne K_3$(otherwise $S-(\{v',v'''\})\cup V(C')\cup \{v_1,v_2\}$ is a TRDS of *G*) and $G\in \Gamma _1^2$. Let $C\in \Gamma _2$ and $v''\in V(C)-S(C)-L(C)$. If $v'\in L(C)$ and $v'''$ is the common neighbor of $v'$ and $v''$, then $V(G)-\{v',v'',v'''\}$ is a TRDS of *G*, which is a contradiction. Hence $v'=v''$. Then when $G[V(C')\cup \{v_2\}]=K_3$, $G\in \Gamma _2^2$; when $G[V(C')\cup \{v_2\}]\ne K_3$, $G\in \Gamma _2^2\cup \Gamma _4^2$. Let *G*[*S*] has the third component $C''$. Then by the claw-freeness of *G*, $v_1$ and $v_2$ have a common neighbor in $C''$ and $N(v_1)\cap V(C'')=N(v_2)\cap V(C'')$. If $C\ne K_2$, then by Claim 1 and Claim 3, $S-\{v'\}$ is a TRDS of *G*, a contradiction. Hence $C=K_2$. Clearly $G[V(C'')\cup \{v_1,v_2\}]\ne K_4$ and $|V(C'')|\ge 3$. If there is a vertex $v''\in N(v_1)\cap V(C'')$ such that $d(v'')=d_{G[V(C'')\cup \{v_1,v_2\}]}(v_1)$, then $S-\{v''\}$ is a TRDS of *G*, which is a contradiction. Hence for any vertex $v''\in N(v_1)\cap V(C'')$, $d(v'')\ge d_{G[V(C'')\cup \{v_1,v_2\}]}(v_1)+1$. It follows that $N(v_1)\cap V(C'')\subseteq S(C'')$. By the claw-freeness of *G*, $N(v_1)\cap V(C'')=S(C'')$ and $C''\in \Gamma _0$. Therefore when $G[V(C')\cup \{v_2\}]=K_3$, $G\in \Gamma _{12}^2$; when $G[V(C')\cup \{v_2\}]\ne K_3$, $G\in \Gamma _5^2$. $\square$

### Claim 6

Let $C,C'$ be two components of *G*[*S*]. If $|N(v_1)\cap V(C)|,|N(v_2)\cap V(C')|\ge 2$ and $|N(v_1)\cap V(C')|=|N(v_2)\cap V(C)|=0$, then $G[V(C)\cup V(C')\cup \{v_1,v_2\}]\in \Gamma _3^2\cup \Gamma _{12}^2$.

### Proof

By the same discussing as the proof of $G[V(C')\cup \{v_2\}]\in \Gamma _2$ in Claim 5, we have $G[V(C)\cup \{v_1\}],G[V(C')\cup \{v_2\}]\in \Gamma _2$. Clearly, at most one of $G[V(C)\cup \{v_1\}],G[V(C')\cup \{v_2\}]$ is isomorphic to $K_3$. When one of them is isomorphic to $K_3$, $G[V(C)\cup V(C')\cup \{v_1,v_2\}]\in \Gamma _{12}^2$; when neither of them is isomorphic to $K_3$, $G[V(C)\cup V(C')\cup \{v_1,v_2\}]\in \Gamma _3^2$. $\square$

### Claim 7

Let *C* be a component of *G*[*S*], $N(v_1)\cap V(C)=\{v'\}$ and $N(v_2)\cap V(C)=\{v''\}$. If $v'=v''$, then $G[V(C)\cup \{v_1,v_2\}]\in \Gamma _2^2\cup \Gamma _8^2\cup \Gamma _{10}^2\cup \Gamma _{12}^2$. If $v'\ne v''$, then $G[V(C)\cup \{v_1,v_2\}]\in \Gamma _6^2$.

### Proof

Denote $G'=[V(C)\cup \{v_1,v_2\}]$. Let $v'=v''$. If $C\in \{K_2,K_3,P_3\}$, then $G'\in \Gamma _{10}^2\cup \Gamma _{12}^2$. Let $|V(C)|\ge 4$. Assume that $C-\{v'\}\not \in \Gamma$ and $S'$ is a $\gamma _{tr}$-set of $C-\{v'\}$. If $|S'|\le |V(C)|-4$, then $S'\cup \{v',v_1,v_2\}\cup (S-V(C))$ is a TRDS of *G*, which is a contradiction. If $N_C(v')\cap S'\ne \emptyset$, then $S'\cup \{v'\}\cup (S-V(C))$ is a TRDS of *G*, which is a contradiction. So $|S'|=|V(C)|-3$ and $N_C(v')\cap S'=\emptyset$. Therefore $d(v')\le 4$. If $N(v')=\{v_1,v_2,u\}$, then $N(u)-(S'\cup \{v'\})\ne \emptyset$. Let $u'\in N(u)-(S'\cup \{v'\})$. Then $V(G)- \{u,u',v'\}$ is a TRDS of *G*, which is a contradiction. Hence $d(v')=4$. Therefore $N_C(v')=V(C)-\{v'\}-S'$. Let $N_C(v')=\{u,u'\}$. Then $uu'\in E(G)$, $d(u),d(u')\ge 3$ and $u,u'\not \in S(C-\{v'\})$. Therefore $V(G)-\{u,u',v'\}$ is a TRDS of *G*, which also is a contradiction. So $\gamma _{tr}(C-\{v'\})=|V(C)|-1$ and $C-\{v'\}\in \Gamma$. Clearly $C-\{v'\}\ne K_3$. When $C-\{v'\}=P_3$, it is easy to check that $G'\in \Gamma _8^2\cup \Gamma _{12}^2$. Let $C-\{v'\}\ne P_3$. Let $v'$ be adjacent to a vertex *u* in $S(C-\{v'\})$. Then by the claw-freeness of *G*, either $v'$ is adjacent to every vertex of $N_{C-\{v'\}}(u)-L(C-\{v'\})$ or $N_C(v')=\{u,u'\}$, where $u'\in N(u)\cap L(C-\{v'\})$. For the former, $G'\in \Gamma _8^2\cup \Gamma _{10}^2\cup \Gamma _{12}^2$. For the latter, $C-\{v'\}\in \Gamma _0$ and $G'\in \Gamma _{10}^2$. Let $v'$ only be adjacent to a vertex in $V(C-\{v'\})-S(C-\{v'\})$. Then $d(v')=3$. When $v'$ is adjacent to one vertex of $L(C-\{v'\})$, $C-\{v'\}\in \Gamma _0$ and $G'\in \Gamma _2^2$. When $v'$ is adjacent to the vertex in $V(C-\{v'\})-S(C-\{v'\})-L(C-\{v'\})$, by the claw-freeness of *G*, $C-\{v'\}\in \Gamma _2$ and $G'\in \Gamma _{12}^2$.

Let $v'\ne v''$. Then by the claw-freeness of *G*, either *G*[*S*] is connected or *G*[*S*] has exactly two components $C,C'$ and $N_{G''}[v_1]=N_{G''}[v_2]$, where $G''=G[V(C')\cup \{v_1, v_2\}]$. For the latter, $C= K_2$. Otherwise there is a vertex $v(\ne v'')$ adjacent to $v'$. When $v'v''\in E(G)$, by the claw-freeness of *G*, $vv''\in E(G)$ and $S-\{v'\}$ is a TRDS of *G*; when $v'v''\not \in E(G)$, since *C* is connected, there is a path $v'v\cdots v''$ and $S-\{v'\}$ also is a TRDS of *G*. By Claim 3, $G[N(v_1)\cap V(C')]\simeq K_{d_{G''}(v_1)-1}$. If there is a vertex *v* in $N(v_1)\cap V(C')-S(C')$, then $V(C)\cup (V(C')-\{v\})$ is a TRDS of *G*, which is a contradiction. Hence $N(v_1)\cap S(C')\ne \emptyset$. If $N(v_1)\cap V(C')\not \subseteq S(C')$, then $S-((N(v_1)\cap V(C')-S(C'))$ is a TRDS of *G*, which is a contradiction. Hence $N(v_1)\cap V(C')\subseteq S(C')$. Clearly $G''\ne K_4$. By the claw-freeness of *G*, $N(v_1)\cap V(C')=S(C')$ and $G-E(C)\in \Gamma _0$. Therefore $G\in \Gamma _6^2$. For the former, let $S'$ be a $\gamma _{tr}$-set of $G-\{v_1v_2\}$. Since $S'$ is a TRDS of *G*, $|S'|\ge n-2$. By $v_1,v_2\in L(G-\{v_1v_2\})$, $v_1,v_2,v',v''\in S'$.

Let $|S'|=n$. Then $G-\{v_1v_2\}\in \Gamma$. When $|V(C)|=2$, $G=C_4$. When $|V(C)|=3$, $G=C_5$. Let $|V(C)|\ge 4$. Then $G-\{v_1v_2\}\in \Gamma _0$ and $G\in \Gamma _6^2$.

Let $|S'|=n-2$. Clearly $|V(C)|\ge 4$ and at least one of $(N(v')-\{v_1\})\cap S', (N(v'')-\{v_2\})\cap S'$ is empty. Let only one of them be empty. Without loss of generality, let $(N(v')-\{v_1\})\cap S'=\emptyset$. Then $d_C(v')\le 2$. Assume that $N_C(v')=\{w,w'\}$. Then $w,w'\not \in S'$, $ww'\in E(G)$ and one and only one of $w,w'$ is 2-degree. Without loss of generality, let $d(w)=2$. Then $(S'\cup \{w'\})-\{v_1,v'\}$ is a TRDS of *G*, which is a contradiction. Thus $d_C(v')=1$. Let $N_C(v')=\{w\}$ and $w'$ be the vertex in $N(w)-S'$. Then $d(w)=2$. Otherwise there is a vertex $w''(\ne v',w')$ adjacent to *w* and $S'-\{v'\}$ is a TRDS of *G*. Let *u* be any vertex in $(N(v'')-\{v_2\})\cap S'$. By Claim 1 and the connectivity of *C*, $d(u)\ge 2$ and when $w'v''\in E(G)$, $w'u\in E(G)$. If $d(v'')\ge 4$, then there are at least two neighbors of $v''$ in $S'$ and $S'-\{v'',v_2\}$ is a TRDS of *G*, which is a contradiction. Hence $d(v'')\le 3$. Assume that $w'\not \in N(u)$ or $w'\in N(u)$ and $d(u)\ge 3$. Then $S'-\{v_2,v''\}$ is a TRDS of *G*, which is a contradiction. Thus $N(u)=\{v'',w'\}$. If $v''w\in E(G)$, then $(S'-\{u,v_2,v''\})\cup \{w,w'\}$ is a TRDS of *G*, which is a contradiction. Thus $v''w\not \in E(G)$ and $d(v'')=2$. If $d(w')\ge 3$, then by the claw-freeness of *G*, $d(u)\ge 3$, a contradiction. Hence $d(w')=2$. It follows that $G=C_7\in \Gamma _6^2$.

Let $(N(v')-\{v_1\})\cap S'=(N(v'')-\{v_2\})\cap S'=\emptyset$. Therefore $d(v'),d(v'')\le 3$ and $v'v''\not \in E(G)$. Assume that $N_C(v')\cap N_C(v'')=\{v\}$. Then $N(v)-\{v',v''\}\ne \emptyset$. Let $u\in N(v)-\{v',v''\}$. Then $S'=V(G)-\{u,v\}$. By the claw-freeness of *G*, $uv'\in E(G)$ or $uv''\in E(G)$. Whether the former or the latter holds, we will get $S'-\{v''\}$ or $S'-\{v'\}$ is a TRDS of *G*, which is a contradiction. Thus either $N_C(v')\cap N_C(v'')=\{u,v\}$ or $N_C(v')\cap N_C(v'')=\emptyset$. For the former, $N[u]=N[v]=\{u,v,v',v''\}$. Then $V(G)-\{v,v_1,v'\}$ is a TRDS of *G*, which is a contradiction. Hence the latter holds and $d(v')=d(v'')=2$. Let $N(v')=\{v_1,u\}$ and $N(v'')=\{v_2,u'\}$. Then $\{u,u'\}=S-S'$ and $uu'\in E(G)$. Clearly, $d(u)=d(u')=2$ and $G=C_6$. $\square$

### Claim 8

Let *C* be a component of *G*[*S*]. If $|N(v_1)\cap V(C)|\ge 2$ and $|N(v_2)\cap V(C)|\ge 2$, then $C\in \Gamma _0$ and $G[V(C)\cup \{v_1,v_2\}]\in \Gamma _7^2$.

### Proof

If $|V(C)|=2$, then $C=K_2\in \Gamma _0$ and $G[V(C)\cup \{v_1,v_2\}]=K_4\in \Gamma _7^2$. Let $|V(C)|\ge 3$. Then by Claim 3, for every vertex *u* of $N(v_1)\cap V(C)$, $d_C(u)\ge |N(v_1)\cap V(C)|$. It follows that $N(v_1)\cap V(C)\subseteq S(C)$. By the same reason, $N(v_2)\cap V(C)\subseteq S(C)$. By the claw-freeness of *G* and the connectivity of *C*, $N(v_1)\cap V(C)=N(v_2)\cap V(C)=S(C)$. Therefore $C\in \Gamma _0$ and $G[V(C)\cup \{v_1,v_2\}]\in \Gamma _7^2$. $\square$

To complete the proof, we discuss the following three cases.

- **Case 1** *G*[*S*] is connected. When $d(v_1)=d(v_2)=2$, by Claim 7, $G\in \Gamma _2^2\cup \Gamma _6^2\cup \Gamma _8^2\cup \Gamma _{10}^2\cup \Gamma _{12}^2$. When only one vertex of $v_1,v_2$ has degree at least 2, by Claim 4, $G\in \Gamma _6^2\cup \Gamma _{9}^2\cup \Gamma _{11}^2$. When $d(v_1),d(v_2)\ge 3$, by Claim 8, $G\in \Gamma _7^2$.
- **Case 2** *G*[*S*] has exactly two components *C* and $C'$. Let $G'=G[V(C)\cup \{v_1,v_2\}]$ and $G''=G[V(C')\cup \{v_1,v_2\}]$.
- **Case 2.1** $v_1v_2$ is a cut edge of *G*. Then $N(v_1)\cap N(v_2)=\emptyset$. By Claims 2, 5 and 6, $G\in (\cup _{i=0}^5\Gamma _i^2)\cup \Gamma _{12}^2$.
- **Case 2.2** $v_1v_2$ isn’t a cut edge of *G*. Then there is a component of *G*[*S*] such that both of $v_1,v_2$ are adjacent to at least one vertex of it. Let *C* be such a component and $N(v_1)\cap V(C')\ne \emptyset$. By Case 1, $G'\in \Gamma _2^2\cup (\cup _{i=6}^{12}\Gamma _i^2)$. It is easy check that $G'\not \in \Gamma _2^2\cup \Gamma _8^2\cup \Gamma _{11}^2$.
  If $|V(C')|=2$, then $C'=K_2$ and $N(v_1)\cap V(C')\subseteq S(C')$. Let $|V(C')|\ge 3$. Obviously, for any vertex *v* of $N(v_1)\cap V(C')$, $d_{C'}(v)\ge |N(v_1)\cap V(C')|$. If there is a vertex *v* in $N(v_1)\cap V(C')-S(C')$, then $S-\{v\}$ is a TRDS of *G*, which is a contradiction. Hence $N(v_1)\cap V(C')\subseteq S(C')$. By the connectivity of $C'$ and the claw-freeness of *G*, for any vertex *v* of $N(v_1)\cap V(C')$, $d_{C'}(v)=|N(v_1)\cap V(C')|$, $C'\in \Gamma _0$ and $N(v_1)\cap V(C')=S(C')$.
- **Case 2.2.1** $G'\in \Gamma _6^2$. Then $d_{G'}(v_1)=2$ or $d_{G'}(v_2)=2$. Without loss of generality, let $N_{G'}(v_1)=\{v_2,v'\}$. Since $N_{G'}(v_1)\cap N_{G'}(v_2)=\emptyset$, by the claw-freeness of *G*, $N(v_1)\cap V(C')=N(v_2)\cap V(C')$. If $C\ne K_2$, then $v'\not \in S(C)$ and $S-\{v'\}$ is a TRDS of *G*, which is a contradiction. Hence $C=K_2$ and $G'=C_4$. Clearly $G''\not \simeq K_4$. When $C'\ne K_2$, both of $v_1,v_2$ are adjacent to every vertex of $S(C')$; when $C'=K_2$, $v_1,v_2$ is adjacent to the same vertex *v* of $C'$. Hence $G\in \Gamma _6^2$.
- **Case 2.2.2** $G'\in \Gamma _7^2$. Then $C\in \Gamma _0$ and both of $v_1,v_2$ are adjacent to every vertex of *S*(*C*). Clearly $G'\ne K_4$. Let $N(v_2)\cap V(C')=\emptyset$. When $C'=K_2$, $G\in \Gamma _5^2\cup \Gamma _8^2$. When $C'\ne K_2$, $G''\in \Gamma _0$ and $G\in \Gamma _8^2$. Let $N(v_2)\cap V(C')\ne \emptyset$. When $C'=K_2$, $|N(v_2)\cap V(C')|=1$. If $N(v_1)\cap N(v_2)\cap V(C')=\emptyset$, then $G\in \Gamma _6^2$; If $N(v_1)\cap N(v_2)\cap V(C')\ne \emptyset$, then $G\in \Gamma _7^2$. When $C'\ne K_2$, $N(v_2)\cap V(C')\subseteq S(C')$. By the claw-freeness of *G*, $N(v_2)\cap V(C')=S(C')$ and by Claim 3, $G[\{v_2\}\cup V(C')]\in \Gamma _2$. Thus $G\in \Gamma _7^2$.
- **Case 2.2.3** $G'\in \Gamma _9^2$. Then $d_{G'}(v_1)=2,d_{G'}(v_2)=3$ or $d_{G'}(v_1)=3,d_{G'}(v_2)=2$. By *G* having exactly two components and Claim 4(1), (2), $C=K_2$ and by the proof of Claim 4(2), $G\in \Gamma _9^2$.
- **Case 2.2.4** $G'\in \Gamma _{10}^2$. Then by the connectivity of *C*, $d_{G'}(v_1)=d_{G'}(v_2)=2$, $v_1,v_2$ have a common neighbor *v* in $G'$ and $C-\{v\}\in \Gamma _0$. Clearly $N(v_2)\cap V(C')=\emptyset$. When $C=P_3$, $G\in \Gamma _{10}^2$. By the claw-freeness of *G*, Claims 1 and 3, $G[\{v_1\}\cup V(C')]\in \Gamma _2\cup P_3$. When $C=K_3$, $G[V(C')\cup \{v_1\}]\ne K_3$ and $G\in \Gamma _{10}^2$. Let $C\ne P_3,K_3$. Assume that *v* is adjacent to each endpoint $u,u'$ of a pendant edge of $C-\{v\}$ and $u\in S(C-\{v\})$, then $(S-\{u',v\})\cup \{v_1\}$ is a TRDS of *G*, which is a contradiction. Hence $N(v)=\{v_1,v_2\}\cup S(C-\{v\})$ and $G\in \Gamma _{10}^2$.
- **Case 2.2.5** $G'\in \Gamma _{12}^2$. Then by the connectivity of *C*, $d_{G'}(v_1)=d_{G'}(v_2)=2$, $v_1,v_2$ have a common neighbor *v* in $G'$ and $G'-\{v_2\}\in \Gamma _1$. Assume that $G'-\{v_2\}\ne P_3$. Let $v'\in V(G)-S(G)-L(G)$. Then $V(G)-\{v,v_2,v'\}$ is a TRDS of *G*, which is a contradiction. Hence, $G'-\{v_2\}=P_3$ and $v\in S(G'-\{v_2\})$. If $N(v_2)\cap V(C')=\emptyset$, then $G[V(C')\cup \{v_1\}]\in \{P_3,K_3\}\cup \Gamma _2$ and $G\in \Gamma _5^2\cup \Gamma _8^2$. Let $N(v_2)\cap V(C')\ne \emptyset$. When $C'=K_2$, $|V(C')\cap N(v_2)|\le 1$. If $N(v_1)\cap N(v_2)\cap V(C')=\emptyset$, then $G\in \Gamma _6^2$. If $N(v_1)\cap N(v_2)\cap V(C')\ne \emptyset$, then $|N(v_1)\cap V(C')|=1$ and $G\in \Gamma _7^2$. Let $C'\ne K_2$. Then by the claw-freeness of *G* and Claim 3, $N(v_2)\cap V(C')=S(C')$ and $G''\in \Gamma _2$. Therefore $G\in \Gamma _7^2$.
- **Case 3** *G*[*S*] has three components $C_1$, $C_2$ and $C_3$. By the claw-freeness of *G*, both $v_1$ and $v_2$ are adjacent to at least one vertex of exactly two components of *G*[*S*]. Without loss of generality, let $N(v_1)\cap V(C_1)\ne \emptyset \ne N(v_1)\cap V(C_2)$ and $N(v_2)\cap V(C_2)\ne \emptyset \ne N(v_2)\cap V(C_3)$. By similarly discussing to the proof of $C'\in \Gamma _0$ in Case 2.2, we have $C_1,C_3\in \Gamma _0$ and when $C_1=K_2$(or $C_3=K_2$), $N(v_1)\cap V(C_1)\subseteq S(C_1)$(or $N(v_2)\cap V(C_3)\subseteq S(C_3)$); when $C_1\ne K_2$(or $C_3\ne K_2$), $N(v_1)\cap V(C_1)=S(C_1)$(or $N(v_2)\cap V(C_3)=S(C_3)$). By the claw-freeness of *G*, $N(v_1)\cap N(v_2)\ne \emptyset$.
- **Case 3.1** $N(v_1)\cap N(v_2)=\{v'\}$. Assume that $C_2\ne K_2$. Then $v'\not \in S(C_2)$ and $S-\{v'\}$ is a TRDS of *G*, which is a contradiction. Hence $C_2=K_2$. Clearly $G[V(C_1)\cup \{v_1\}]$ and $G[V(C_3)\cup \{v_2\}]$ can’t be isomorphic to $K_3$ at the same time. Let one of them be isomorphic to $K_3$. Without loss of generality, let $G[V(C_1)\cup \{v_1\}]\cong K_3$. If $C_3=K_2$, then $G\in \Gamma _{12}^2$; if $C_3\ne K_2$, then $G\in \Gamma _8^2$. Let neither of them be isomorphic to $K_3$. If one of $C_1,C_3$ is $K_2$, then $G\in \Gamma _5^2$; if neither of $C_1,C_3$ is $K_2$, then $G\in \Gamma _8^2$.
- **Case 3.2** $|N(v_1)\cap N(v_2)|\ge 2$. By Claim 8, $C_2\in \Gamma _0$ and $G[V(C_2)\cup \{v_1,v_2\}]\in \Gamma _7^2$. Clearly $C_2\ne K_2$ and $G[V(C_1)\cup \{v_1\}],G[V(C_3)\cup \{v_2\}]$ can’t be isomorphic to $K_3$ at the same time. By similarly discussing as Case 3.1, we have when one of them is isomorphic to $K_3$, $G\in \Gamma _8^2\cup \Gamma _{12}^2$ and when neither of them is isomorphic to $K_3$, $G\in \Gamma _5^2\cup \Gamma _8^2$. $\square$

## Conclusions

The study focuses on the total restrained domination in claw-free graphs. Firstly, in the course of analysis, I construct 12 kinds of connected claw-free graphs with order *n* and the total restrained domination number $n-2$. Let $\Gamma ^2$ denote the set of these claw-free graphs. Secondly, by discussing all possible cases of the induced subgraph of the minimum total restrained dominating set, I show that if the total restrained domination number of a connected claw-free graph with order *n* is $n-2$, then the graph must belong to $\Gamma ^2$. In a word, as for a connected claw-free graph with order *n*, the conclusion gives a method to judge whether the total restrained domination number of it is $n-2$. Further research can focus on the construction of connected claw-free graphs with order *n* and total restrained domination number $n-3$ and characterize these graphs, although it may be very difficult and complicated.
